# An improved method for extraction of polar and charged metabolites from cyanobacteria

**DOI:** 10.1371/journal.pone.0204273

**Published:** 2018-10-04

**Authors:** Charulata B. Prasannan, Damini Jaiswal, Rose Davis, Pramod P. Wangikar

**Affiliations:** 1 Department of Chemical Engineering, Indian Institute of Technology Bombay, Powai, Mumbai, India; 2 DBT-Pan IIT Center for Bioenergy, Indian Institute of Technology Bombay, Powai, Mumbai, India; 3 Wadhwani Research Center for Bioengineering, Indian Institute of Technology Bombay, Powai, Mumbai, India; CEA-Saclay, FRANCE

## Abstract

A key requirement for ^13^C Metabolic flux analysis (^13^C-MFA), a widely used technique to estimate intracellular metabolic fluxes, is an efficient method for the extraction of intermediate metabolites for analysis via liquid chromatography mass spectrometry (LC/MS). The ^13^C isotopic labeling results in further distribution of an already sparse pool of intermediate metabolites into isotopologues, each appearing as a separate chromatographic feature. We examined some of the reported solvent systems for the extraction of polar intracellular metabolites from three strains of cyanobacteria of the genus *Synechococcus*, viz., *Synechococcus* sp. PCC 7002, *Synechococcus elongatus* PCC 7942, and a newly isolated *Synechococcus elongatus* PCC 11801 (manuscript under review). High resolution-LC/MS was used to assess the relative abundance of the extracted metabolites. The different solvent systems used for extraction led to statistically significant changes in the extraction efficiency for a large number of metabolites. While a few hundred m/z features or potential metabolites were detected with different solvent systems, the abundance of over a quarter of all metabolites varied significantly from one solvent system to another. Further, the extraction methods were evaluated for a targeted set of metabolites that are important in ^13^C-MFA studies of photosynthetic organisms. While for the strain PCC 7002, the reported method using methanol-chloroform-water system gave satisfactory results, a mild base in the form of NH_4_OH had to be used in place of water to achieve adequate levels of extraction for PCC 7942 and PCC 11801. While minor changes in extraction solvent resulted in dramatic changes in the extraction efficiency of a number of compounds, certain metabolites such as amino acids and organic acids were adequately extracted in all the solvent systems tested. Overall, we present a new improved method for extraction using a methanol-chloroform-NH_4_OH system. Our method improves the extraction of polar compounds such as sugar phosphates, bisphosphates, that are central to ^13^C-MFA studies.

## Introduction

Among the omics-based approaches, metabolomics provides cellular information that is much closer to the phenotype of the organism. To exemplify, genomics can unravel the cell’s capability to perform a certain function while transcriptomic and proteomic studies assess the readiness to execute the function. Metabolomics on the other hand has the capability to provide the phenotypic endpoint at least for the metabolic functions of the cell [1,2]. While recent metabolomics studies have discovered new metabolites and biochemical pathways of physiological relevance, there are many more which are yet to be detected and quantified [3–8]. Time resolved metabolite profiling with absolute quantification is being increasingly used to understand the dynamics of enzyme kinetics and metabolic reaction networks. These studies have become feasible with the recent advances in high throughput mass spectrometry that enables precise and accurate characterization of intracellular metabolites. The metabolite profiling furnishes a static snapshot of the metabolites in the cell while metabolic flux analysis (MFA), a complementary approach to untargeted metabolomics, yields metabolic context of the cells with respect to the environmental conditions. The ^13^C MFA approach provides dynamic information about the rate of metabolic reactions of an organism using isotopic labeling data and a network model [9–12], which support the metabolic engineering efforts. Isotopic non stationary MFA (INST-MFA) is a relatively new approach which provides highly resolved flux estimates for complex systems such as animal cell cultures, photoautotrophic organisms and plants [13]. Non stationary MFA entails the quantitation of intermediate metabolites that are typically less abundant apart from being labile [14,15]. The experimental design comprises of a tracer experiment which follows the ^13^C isotopic labeling of intermediate metabolites in a short time scale, typically over seconds or minutes. Therefore, rapid sampling, quenching and extraction, identification and quantification of metabolites, and the ^13^C label incorporation are the essential steps for ^13^C-MFA studies. Although proper sample preparation is an integral part of all omics approaches, it is indispensable for ^13^C-MFA or fluxomics where the metabolite pools get further divided into mass isotopologues. The choice of extraction method can drastically impact the measurement of low abundance mass isotopologues [16] and can lead to erroneous prediction of fluxes. Extraction protocols are evaluated based on the number of features detected, the intensity of features detected, the ability to extract different classes of metabolites, regulating the chemical/ biochemical degradation of metabolites, and reproducibility.

Methods for extraction of intracellular metabolites have evolved concomitantly with the advancements in the LC/MS hardware and data analysis software. The extraction methods vary in terms of the nature of extraction solvent, composition of solvent mixtures, added acid or alkali, and temperature of extraction. The use of hot water and boiling ethanol to extract polar metabolites with excellent reproducibility was reported in the 1940’s and 1970’s respectively [17,18]. Although this method was not considered to be suitable for phosphorylated metabolites, nucleotides, tricarboxylic acids, and other thermo-labile compounds, it is still used to extract certain intracellular metabolites [19,20]. With the presumption that cold extraction will lower the risk of metabolite degradation, the cold methanol method was developed in early 1990 and is used extensively even today [19]. The acid and alkali based methods were developed around the same time, during the late 80’s and early 90’s, when the utilization of extremes of pH at cold conditions was used to disrupt the cell wall and inactivate the enzymes for efficient extraction of metabolites [21,22]. These methods are considered with caution because only the acid or alkali stable compounds can be analyzed [19,23]. The use of methanol-chloroform-water was first reported for the extraction of total lipids from tissue cells in late 50’s [24]. This ternary system was first employed in a microbial system, in the year 1992, for the extraction of polar and non-polar metabolites [23]. Since then this solvent system has been reported in a number of studies [25,26]. Diverse solvent systems were first examined, in the year 2007, for efficient extraction of nucleotide triphosphates (NTPs) from *E*. *coli* with the conclusion that acidic acetonitrile-methanol system is optimal for this organism [27]. However, it led to poor recovery of metabolites when applied to a yeast system [16]. The completeness of extraction will depend on the susceptibility of cell for lysis as well, which varies in prokaryotes vs. eukaryotes and/or gram negative vs. gram positive organisms, and therefore variations are reported when a method is used in a different organism. Thus, a variety of extraction methods have been reported although the efficiency of these methods depends on the biological systems in which they are applied. The ongoing area of investigation in the field of metabolite extractions is to identify the best extraction procedures as a function of sample type and or compounds of interest [28].

Cyanobacteria, a group of prokaryotes capable of oxygenic photosysntheis, are considered to be a promising resource for the production of biofuels and commodity chemicals [29,30]. While a number of solvent mixtures such as methanol-water [31–34], methanol-ammonium formate [35], methanol-chloroform-water [36,37] and methanol-isopropanol-water [38] have been reported for cyanobacteria and/or micro algae, a systematic comparison of efficiency of extraction has not been reported for these photoautotrophs. Although the monophasic solvent system of methanol-water has been widely used, the co-extraction of pigments into the aqueous phase can confound the identification and quantification of the metabolites of interest. Although the use of acid or addition of a buffer at low pH can alleviate this problem to a certain extent, several classes of metabolites of interest in ^13^C-MFA studies show poor recovery under acidic conditions [39,40]. Therefore, the main objective of this study was to devise an extraction method that would enable the identification and quantification of intracellular metabolites from glycolysis pathway, and the TCA and calvin cycles which are essential in ^13^C-MFA studies. Thus, in this study, we compare the effect of addition of water to the extraction solvent and the use of a weak base for phase separation on three cyanobacterial strains of the genus *Synechococcus*. Here, we present an efficient and improved metabolite extraction method which allows the detection of several classes of compounds, has improved yields for many metabolites, and is reproducible. The overall analysis of the methods was based on both targeted and untargeted set of metabolites. With the use of a methanol-chloroform-NH_4_OH extraction mixture we can effectively quantify the mass isotopologue distributions (MIDs) of many sugar phosphates which in turn will provide better flux estimates.

## Experimental section

### Chemical materials

All chemicals used were purchased from Sigma Aldrich (St. Louis, MO) and Merck (Burlington, MA).

### Strains and culture conditions

Cyanobacterial strains *Synechococcus sp*. PCC 7002 (henceforth referred to as PCC 7002), *Synechococcus sp*. PCC 7942 (PCC 7942), and *Synechococcus sp*. PCC 11801 (PCC 11801) were grown in Erlenmeyer flasks with orbital shaking of 120 rpm under continuous light. The strain PCC 7002 was grown in BG11+ASNIII (50% v/v) medium supplemented with 10μg L^-1^ vitamin B_12_ at 38°C and 300 μmol photons m^-2^ s^-1^. The strains PCC 7942 and PCC 11801 were grown in BG11 medium at 30°C, 150 μmol photons m^-2^ s^-1^ and 38°C, 400 μmol photons m^-2^ s^-1^, respectively. The illumination was provided using PhotoBioSim solid state lamps. A 20 mL volume of cell suspension was used for metabolite extractions from the exponentially growing cells at an optical density (at 730 nm) of ∼0.7.

### Metabolite extraction

Three extraction methods using biphasic solvent system were used in this study; Method 1 (methanol + chloroform + water), Method 2A ((80:20 methanol-water)+ chloroform + water) and Method 2B ((80:20 methanol-water) + chloroform + NH_4_OH). In method 2B varying concentrations of NH_4_OH solution in water, 0.2 M, 0.4 M, 0.8 M and 1.6 M were used to optimize the method for each strain. The cell suspension was filtered through a 0.8 μm nylon membrane (Whatman, Maidstone, UK) in a filtration assembly. The filter with cells was quickly placed in 1.6 ml cold methanol (100%) or methanol-water mixture (80:20 v/v) facing cell side down in a round bottom 100 mm dish to quench the metabolism. The mixture was incubated at -80°C for 1 hour following which the cell-solvent mixture was removed and placed in a 15 mL centrifuge tube. The cells sticking to the filter were then scraped off with chloroform (2.4 mL) and mixed with the cell-solvent mixture. The mixture was vortexed for 20 minutes under cold conditions (vortexed for 30s and kept on ice for 1 minute and the cycle repeated). Two mL of MilliQ water or NH_4_OH solution in water (at concentrations of 0.2 M, 0.4 M, 0.8 M, 1.6 M) was added into the mixture to separate the phases. The mixture was vortexed for 10 minutes, centrifuged for 15 minutes at 3200g at 4°C to obtain two separate phases and the top aqueous-rich layer was collected (approximately 3 mL), evaporated to dryness and stored at -80°C until further analysis. The stored pellets were reconstituted in 100 μL of 50/50 methanol-water mixture and filtered before injecting on LC/MS.

### LC/MS analysis

UHPLC (Shimadzu, Nexera LC-30 AD, Singapore) coupled with a Triple TOF 5600+ mass spectrophotometer (SCIEX, Framingham, MA) was used for all the LC/MS analysis. Chromatographic separation was achieved by injecting 10 μL sample on a C-18 synergi-hydro RP column (Phenomenex, Torrance, CA) as described previously using an ion-pairing reagent [41]. The mobile phases used were, solvent A: 10 mM tributylamine and 11 mM acetic acid and solvent B: methanol. The gradient program was as follows, t = 0 min, 0% B; t = 2 min, 0% B; t = 8 min, 35% B; t = 10.5 min, 35% B; t = 15.5 min, 90% B; t = 20.5 min, 90% B; t = 22 min, 0% B at a flow rate of 0.3 mL/min and column temperature 25ºC. The MS data was collected in negative ion mode with an ion spray voltage of 4500 V and interface heater temperature of 450ºC. The ion source gas 1 and gas 2 (GS1 and GS2) and curtain gas were set at 40, 40, and 35 respectively.

### Data analysis

Data presented in this study comprised of two biological replicates and two/three technical replicates making it n = 6 injections for strain PCC 11801 and n = 4 for strains PCC 7002 and PCC 7942. Data analysis for untargeted metabolites was performed using the XCMS online software available at https://xcmsonline.scripps.edu/ [42–47]. The software parameters for pairwise analyses were set as follows, centwave settings for feature detection (delta m/z = 25 ppm, minimum peak width = 5 s and maximum peak width = 60 s); S/N ratio 6; and m/z difference 0.01. The obiwarp setting for retention time correction where the alignment parameters were fixed at bandwidth or retention time variation of 5 s, mz-width of 0.05, and minfrac 0.5. The metabolic features were defined as ions with a unique m/z and retention time and their identity was based on putative matches from the Metlin Database [43]. An in-house database created using the available standards was used to confirm the putative matches [48]. The fold change and the peak intensity output for the significant m/z features at p value ≤ 0.05 was used for comparison between the extraction methods. Within pairwise data analysis option of XCMS online, an unpaired parametric t-test (Welsh t-test) was used to obtain significant m/z features [49]. ANOVA analysis was performed on all the peak areas calculated at various concentrations of NH_4_OH. Cloud plots obtained from the analysis were used to represent the change in features between the two conditions [46]. The extracted ion chromatograms were visualized for the putative metabolites using commercial softwares, Peakview 2.2 and MasterView 1.0 (SCIEX, Framingham, MA). The precursor ions for a targeted set of metabolites were integrated using MultiQuant 3.0.1 (SCIEX, Framingham, MA).

## Results and discussion

**Fig 1:**
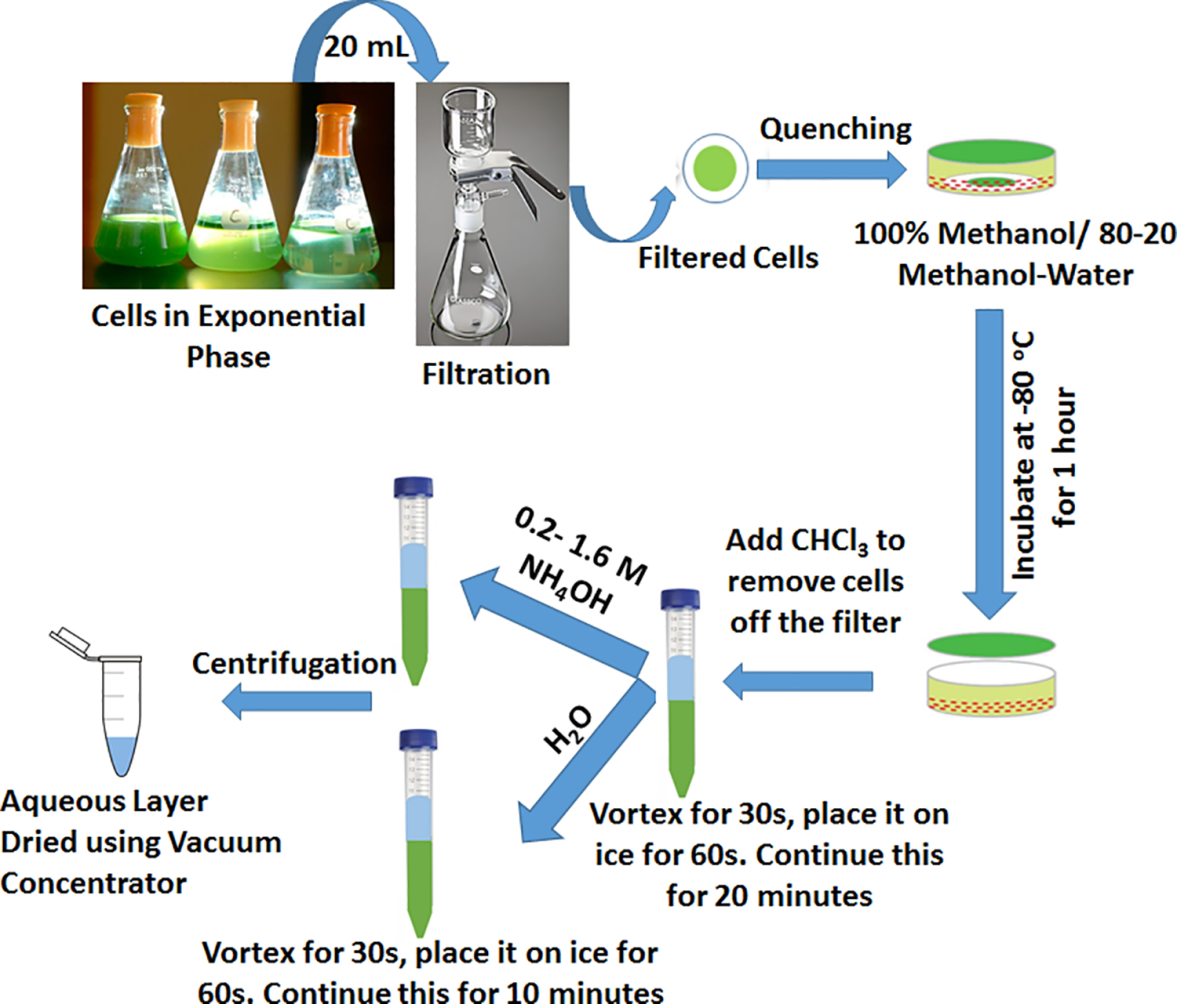
Workflow for the extraction of metabolites from cyanobacteria. Cyanobacterial cells were grown under autotrophic conditions to an optical density of 0.6–0.8. Twenty milliliter culture volume was filtered through a 0.8 μm nylon membrane in the presence of light. The filtered cells were placed in chilled solvent (100% methanol or methanol-water mixture in 80:20 proportion), with cell side facing down, and incubated at -80° C for 1 hour. Chloroform solution was added to remove the cells off the filter. The cells suspension in methanol-chloroform mixture was vortexed well to ensure complete extraction. The mixture is vortexed vigorously for 30s and placed on ice for one minute to minimize the degradation of metabolites. Water or various concentrations of NH_4_OH solution was then added to break the solvent phase. The mixture was vortexed well for 10 minutes by keeping intermittently on ice. The mixture was centrifuged for 15 minutes at cold conditions to separate the phases. The upper aqueous layer was collected and dried using a vacuum centrifuge for metabolite analysis.

Increasing concerns over the sustainability of petroleum-derived fuels and commodity chemicals have led to the exploration of renewable sources for these products that touch our lives every day. Among the various biological routes, photosynthetic microbial cell factories using engineered cyanobacteria offer promise for the production of a variety of chemicals from carbon dioxide feedstock. We argue that the success of this approach will depend on the availability of robust strains of cyanobacteria and a detailed understanding of the metabolism of the host organism. Commonly used model strains for these engineering efforts include *Synechococcus elongatus* PCC 7942, *Synechocystis* PCC 6803, *Synechococcus* sp. PCC 7002 and *Anabena* sp. PCC 7120 [50]. Towards the quest of a robust host, a fast growing, salt tolerant, temperature and CO_2_ tolerant local strain (PCC 11801) was isolated (manuscript under revision) and is being used for metabolic engineering efforts. In order to understand the carbon flow mechanism, we use the isotopically transient labeling data of intracellular metabolites to estimate fluxes using non-stationary ^13^C-MFA approach. To obtain accurate transient labeling data, it is required to quantitate the mass isotopologues which can be present in very minute quantities and therefore the efficiency of extraction is extremely important. To perform isotopic non-stationary ^13^C MFA for the strains PCC 11801 and PCC 7942, we started out with a reported extraction method (method 1) which has also worked in our hands for the strain PCC 7002 [10]. This method primarily involves quenching of filtered cells with cold methanol and extraction in methanol-chloroform-water mixture in 1:2:1 ratio (refer to Fig 1 and the methods section). The cyanobacterial cultures grown under photoautotrophic conditions were filtered rapidly in the presence of light to ensure integrity of the light sensitive metabolites and then the filter paper was transferred to cold methanol to quench the metabolism [10,39]. The cells were removed from the filter paper using chloroform solution and the cell suspension in methanol-chloroform mixture was vortexed to break the cells and extract the metabolites. Water was added to separate the phases, and the upper aqueous layer was used for the analysis of polar or charged metabolites. While the method worked well for PCC 7002, the extraction efficiency was not satisfactory for the strains PCC 11801 and PCC 7942 (S1–S3 Figs). Therefore, there was a need for a modified method for metabolite extraction from these strains.

**Fig 2:**
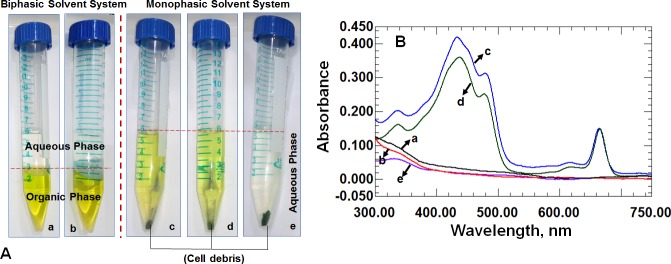
Comparison of aqueous phase between the monophasic and biphasic solvent system. Solvent systems used in this study are a: Method 1 (methanol-chloroform-water), b: Method 2B (80–20 methanol-water-chloroform: 0.2 M NH_4_OH) c) Acetonitrile-methanol-water 40:40:20 d) Methanol-water (80–20) and e) Methanol-ammonium formate buffer pH 3.4. Panel A: A photograph of the cyanobacterial extracts depicting the extraction of pigments in the lower chloroform phase with a clear upper aqueous layer in the biphasic solvent systems a and b. Co-extraction of pigments into the aqueous phase is visible in monophasic systems c and d. Panel B: Absorbance spectrum of the aqueous layer for the various solvent systems tested. Peaks for chlorophyll a, carotenoids and chlorophyll b are seen at wavelength 440 nm, 470 nm and 670 nm respectively for the solvent system c and d. For the biphasic solvent system studied, a and b, and the monophasic system at low pH, e, the pigments are not extracted into the aqueous phase thus decreasing the complexity of the mixture.

In method 1, the process of filtration and quenching was completed in less than 10 seconds and thus the method was considered suitable for time course measurements during isotopic ^13^C carbon labeling experiments. Direct quenching of the dilute cyanobacterial cultures with methanol-water mixture results in low extraction efficiency possibly due to leaching of metabolites while direct centrifugation takes minimum of 2 min before quenching the cell pellet with a solvent and thus not considered suitable for time course measurements [39]. Therefore, while modifying the method, we primarily worked on the nature of solvent keeping the filtration and quenching steps unaltered. While several monophasic solvent systems have been reported for extraction of metabolites from *E*. *coli* and a few cyanobacterial strains, we observed co-extraction of pigments with the monophasic systems (Fig 2). Five different solvent systems (two biphasic and three monophasic) were tested on the strain PCC 11801. The biphasic systems used were those reported in this study a) Method 1 and b) Method 2B with 0.2 M NH_4_OH while the monophasic systems were based on literature c) acetonitrile:methanol:water (40:40:20) [27], d) methanol-water (80:20) (ref) and e) methanol-ammonium formate buffer at pH 3.4 [35]. We observe significant extraction of chlorophyll and carotenoids with the methods c and d. In an absorbance spectrum for solvent system c and d, the peaks at 440 nm and 470 nm have been attributed to chlorphyll and carotenoids, respectively (Fig 2B) [51]. The concentration of the pigments in these cells is very high (~1% dry cell weight) compared to the intracellular metabolites (present in nanomolar quantities) and therefore the presence of pigments interferes with the identification and quantitation of metabolites of interest (data not shown). Moreover, the injection of concentrated solutions of pigments into the mass spectrometer can have adverse effects on the sensitivity of the mass detector over time. In both the biphasic solvent systems (a and b), a clear aqueous layer with metabolites, an organic layer where pigments are extracted, and an interface with cell debris were observed. A number of pigments that are typically present in cyanobacteria get extracted into the chloroform phase thereby reducing the complexity of the aqueous phase for the analysis of metabolites of interest. This phase can be analyzed separately to detect pigments and other non-polar compounds using an apt chromatographic method. Although the methanol/ammonium formate buffer system at pH 3.4 minimizes extraction of pigments into the aqueous layer, we did not use this method since some of the sugar phosphates are known to be labile at low pH [52].

We chose to introduce two modifications, viz., quenching with methanol-water mixture rather than just methanol (method 2) and the addition of mildly basic NH_4_OH solution for phase separation in the last step in place of distilled water (method 3). The use of mild base is known to improve the extraction of negatively charged intermediates into the aqueous layer [53] while the use of methanol-water mixture during quenching has been widely reported.

### Data analysis

The samples were injected on HR-LC/MS/MS and the mass spectrometry results were analyzed for the number of features detected, quality and intensity of the peaks, and reproducibility of results. The three methods were compared based on fold change in peak intensity of untargeted metabolites via XCMS online [42–47] and targeted metabolites with MultiQuant 3.0.1. Targeted metabolites comprised those that are typically used in isotopic non-stationary ^13^C-MFA. The XCMS online parameters were modified as detailed in the methods section. Pairwise comparison of the extraction methods for these strains revealed hundreds of features (refer to S1 File for complete list of features detected, the fold change, and the p-value). Based on the statistical analysis performed using the XCMS online, we limited our analysis to significant features with p value ≤ 0.05 and a fold-change of > 1.5 fold between the two extraction methods.

### Effect of addition of water in the quenching solution

**Fig 3:**
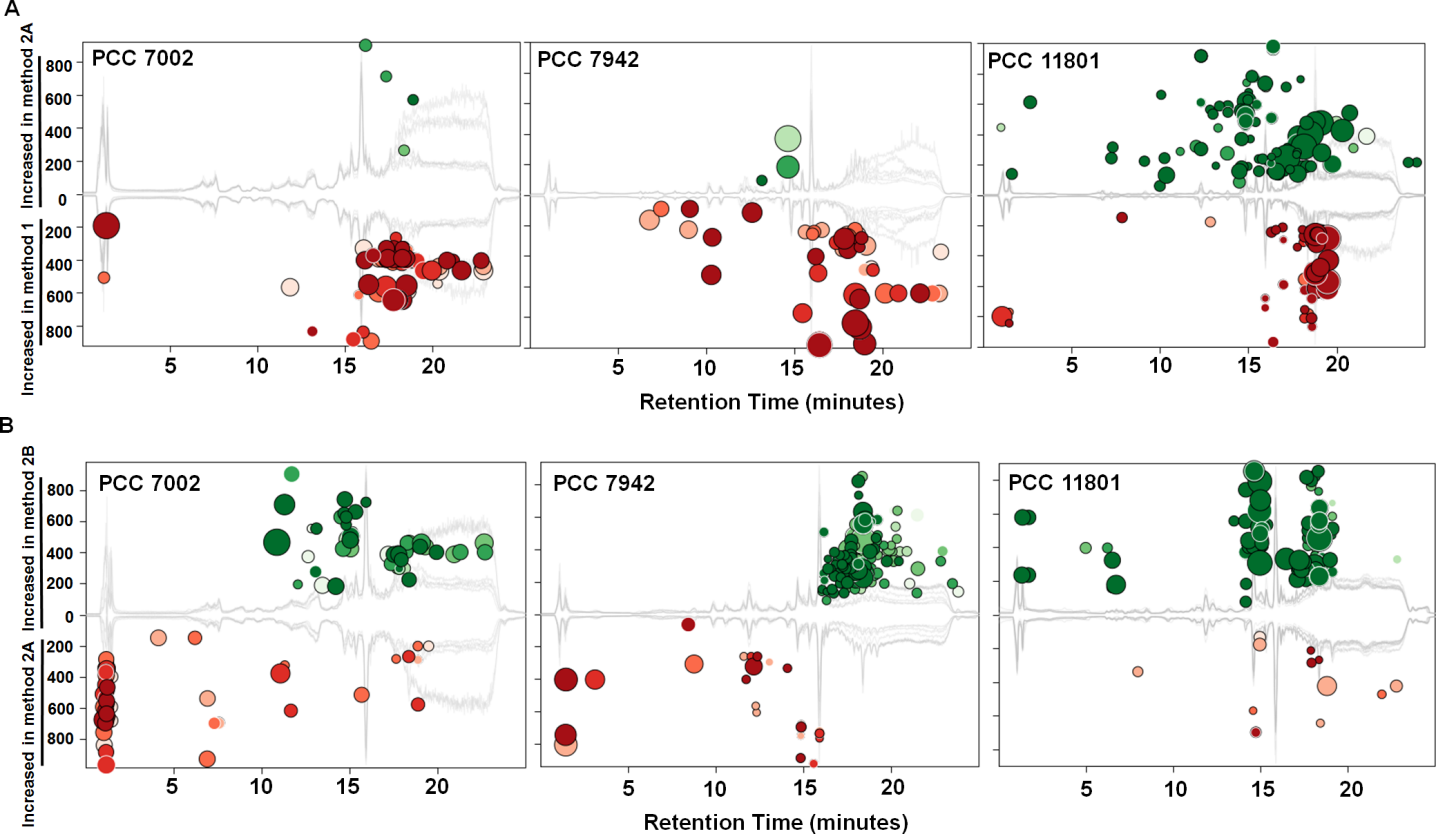
Cloud plots representing the m/z features which display statistically significant differences in peak intensities between the extraction conditions. Panel A: Comparison of features obtained with method 2A vs. method 1 in each of the three cyanobacterial strains. Panel B: Comparison of features obtained with method 2B vs method 2A. For brevity, only those features are displayed in cloud plots that show a fold change ≥ 1.5 and a p-value of ≤ 0.01. The intensity of the color represents the p-value (darker color for lower p-value) while the size of the bubble corresponds to the fold change (larger bubble for greater fold change). This figure was obtained using the cloud plot feature [46] from the XCMS online software using our data.

In an untargeted analysis with XCMS online, method 2 that uses quenching with methanol-water mixture showed improved abundance for many features in the strain PCC 11801 (Table 1, Fig 3A). A significant improvement in the peak quality and intensity was also observed with method 2, especially in strain PCC 11801 (S4 Fig). These features were spread throughout the chromatographic profile (Fig 3A) and included metabolites such as 3 phosphoglyceric acid (3-PGA), glucose 6-phosphate (G6P), sedoheptulose 7-phosphate (S7P), uridine diphosphate glucose (UDP-glucose), and adenosine diphosphate glucose (ADP-glucose), that are normally analyzed for ^13^C-MFA in cyanobacteria. On the other hand, a number of features showed lower abundance with method 2 for the strain PCC 7002 (Table 1, Fig 3A). On a positive note, these features did not include the metabolites that are used for ^13^C-MFA but rather were found at retention time greater than 15 minutes in the chromatographic profile. A number of these were putatively identified as fatty acids. It is known that extraction of fatty acids into the aqueous phase is favored at a basic pH [54,55]. We believe the change in pH with the inclusion of 80–20 methanol-water adversely affects the extraction of fatty acids in the strain PCC 7002.

**Table 1:** Effect of quenching with methanol-water mixture on metabolite extraction: Comparison of efficiency of extraction between methods 1 and 2A^a^ for cyanobacterial strains via an untargeted analysis using XCMS online^b^.

Cyanobacteria Strain Name	Number of detected features	Features with fold-change >1.5 and p-value < 0.05^c^^,^ ^d^	
		Increased intensity with method 2A	Increased intensity with method 1
*Synechococcus* sp. PCC 7002	759	16	153
*Synechococcus elongatus* PCC 7942	359	13	98
*Synechococcus elongatus* PCC 11801	593	155	108

^a^Analysis of m/z features or potential metabolites from cyanobacterial strains using extraction methods, *viz*., method 1 (100% methanol-chloroform-water); method 2A ((80–20 methanol-water)-chloroform-water); and method 2B ((80–20 methanol-water)-chloroform-NH_4_OH) solvent systems.

^b^LC/MS data was analyzed using XCMS online [42–47].

^c^Statistical analysis was based on the number of replicate measurements for each condition n = 4 comprising of two biological replicates with 2 technical replicates of each for the strains PCC 7002 and PCC 7942

^d^Statistical analysis was based on the number of replicate measurements for each condition n = 6 comprising of three biological replicates with 2 technical replicates of each for the strain PCC 11801

In case of the strain PCC 7942, method 2 did not lead to a significant increase in the number of peaks detected (359 features in total, Table 1). Although we did see an increase in intensity for a few metabolites (Fig 3A) many of the metabolites essential for MFA studies were completely missing in PCC 7942, which necessitated further modification of the extraction method. Our study highlights a very important aspect in the field of metabolomics. Two extraction protocols, method 1 and method 2A which worked efficiently for strains PCC 7002 and PCC 11801 respectively, did not give satisfactory extraction for the strain PCC 7942. Thus, results on comparative metabolome analysis of different cyanobacterial strains may be confounded by the strain-to-strain differences in the extraction efficiency.

### Effect of addition of NH_4_OH during phase separation

**Fig 4:**
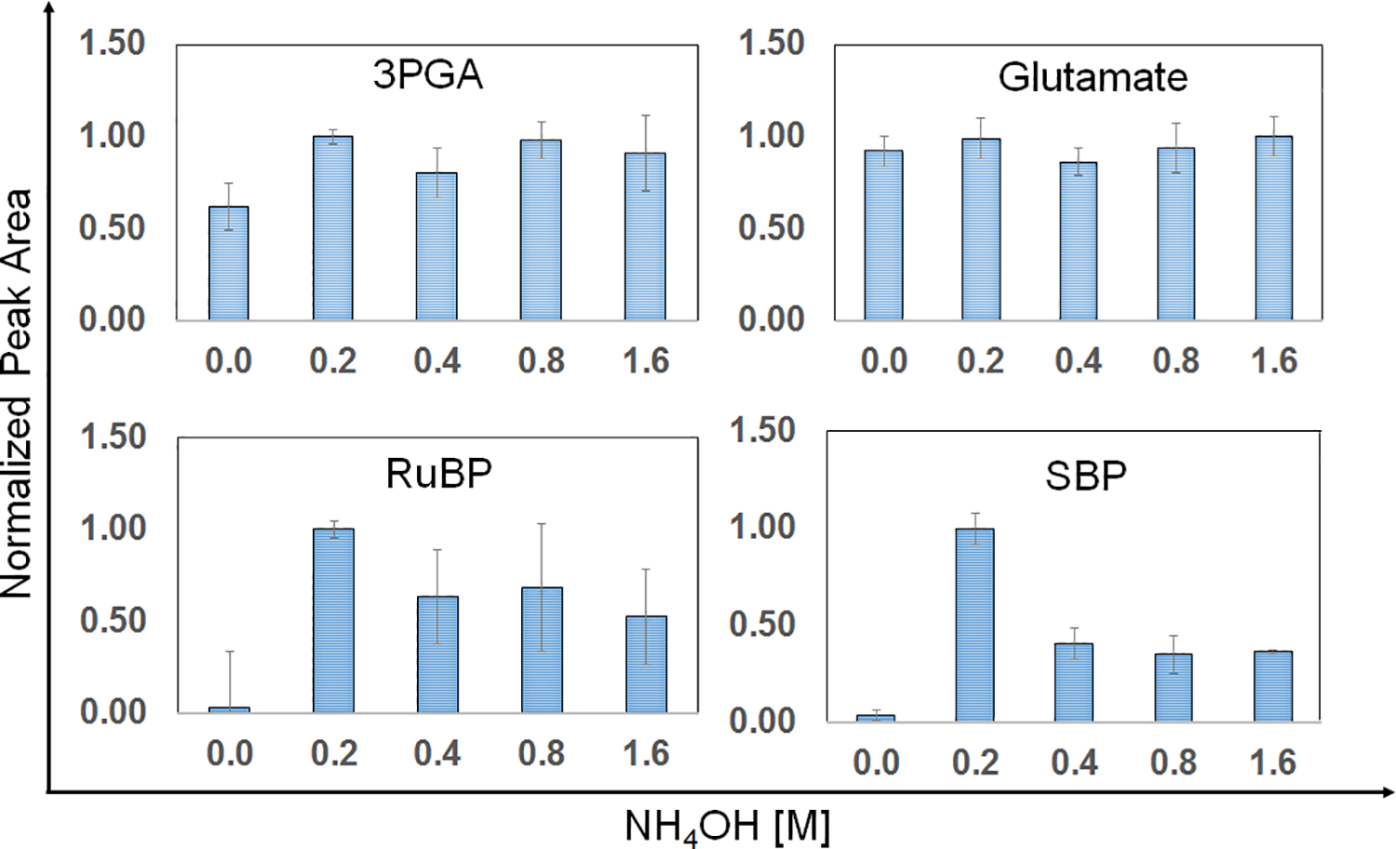
Effect of varying concentrations of NH_4_OH on extraction of a representative set of metabolites from the strain *Synechococcus elongatus* PCC 11801. The normalized peak area for the four metabolites (3PGA, Glutamate, RuBP, and SBP) plotted with respect to the NH_4_OH concentration. Analysis of this data and that for other metabolites of interest led us to choose 0.2 M NH_4_OH solution for PCC 11801. Such analysis was also extended to other strains and we find addition of 0.4 M NH_4_OH suited the best for strains PCC 7002 and PCC 7942. Ribulose 1, 5-bisphosphate (RuBP); 3 Phosphoglyceric acid (3-PGA); Sedoheptulose bisphosphate (SBP).

In a study on *E*. *coli* where various solvent systems were examined, it was concluded that acetonitrile-methanol-water (40:40:20) gave superior yields for nucleotide triphosphates (NTPs) [27]. However, a closer survey of the data shows that the use of weak base NH_4_OH may improve extraction of a broad range of compounds and not just the NTPs which was the focus of that study. We find that method 2B shows improved extraction efficiency in all three strains for the compounds of interest for ^13^C-MFA studies. In order to choose the most effective concentration of NH_4_OH, extraction efficiencies were compared at 4 different concentrations of NH_4_OH in the range of 0.2–1.6 M. A significant effect was observed on different classes of metabolites and one such analysis using the strain PCC 11801 has been shown in Fig 4. For metabolites such as 3-PGA, a significant increase in the intensity was observed with the addition 0.2 M NH_4_OH solution with no further increase with the use of higher concentrations of the base. Amino acids such as glutamate did not show any change with the addition of NH_4_OH. Sugar bisphosphates such as Ribulose bisphosphate (RuBP) and Sedoheptulose Bisphosphate (SBP) which were barely detected without using NH_4_OH could be satisfactorily quantified with method 3. In case of Acetyl Coenzyme A (Acetyl-CoA), the intensity decreased significantly with the addition of NH_4_OH and more so at higher concentrations (data not shown). Acetyl-CoA is known to be stable in the acidic conditions but not under basic conditions (Acetyl coenzyme A sodium salt, A2056, Product Information Sheet, Sigma Aldrich). This analysis with varying concentrations of NH_4_OH was performed on all the strains (S2 File). Based on the number of significant features that showed an increase in intensity, a concentration of 0.4 M NH_4_OH was best suited for the strains PCC 7002 and PCC 7942 (Table 2) while 0.2 M of NH_4_OH was suitable for the strain PCC 11801.

**Table 2:** Effect of addition of mild base NH_4_OH on extraction efficiency: Comparison of efficiency of extraction between methods 2A and 2B for cyanobacterial strains via an untargeted analysis using XCMS online^a^.

Effect of addition of NH_4_OH				
Strain Name	Concentration of NH_4_OH^b^	Number of detected features	Features with fold change >1.5 and p-value < 0.05	
			Increased intensity with method 2B	Increased intensity with method 2A
*Synechococcus* sp. PCC 7002	0.4 M	742	176	112
*Synechococcus elongatus* PCC 7942	0.4 M	647	247	49
*Synechococcus elongatus* PCC 11801	0.2 M	995	175	35

^a^Refer to footnotes to Table 1 for additional details.

^b^NH_4_OH concentrations of 0.2, 0.4, 0.8 and 1.6 M were tested for each strain and the concentration that gave the best results reported here and then used for subsequent analysis.

When compared to using water for phase separation of aqueous and organic layer, the use of NH_4_OH allowed the extraction of many more metabolites into the aqueous phase. This was most prominent for the strain PCC 7942 where the number of features detected nearly doubled (from 359 to 647 detected features, Table 1 and Table 2) with approximately 40% (247 out of 647 features, Table 2) of the metabolites detected had a significant increase in the intensity. For a targeted set of metabolites, the changes observed in intensity (Increased, Decreased, or No Change) for the three strains and the respective fold-change with the variation in extraction solvent are shown in Table 3. Many of the multimer ion adducts such as the dimer adducts of 3-PGA, phosphoenolpyruvate (PEP) and RuBP were also detected in the samples with significant increase in intensity in method 3 (Table 3). The putative features identified based on METLIN database using XCMS online are listed (S1 Table) with the respective fold change detected based on the comparison of methods. Most of the putative features also had an increase in intensity with the use of base in the extraction solvent.

**Table 3:** Effect of quenching with methanol-water mixture (method 2A) and addition of NH4OH during phase separation (method 2B) on a representative set of targeted metabolites that are typically used in ^13^C-metabolic flux analysis.

Metabolite List^c^	m/z	RT^b^	*Synechococcus elongatus* PCC 11801		*Synechococcus elongatus* PCC 7942		*Synechococcus* sp. PCC 7002	
			^a^Observed changes in the intensity with respective fold change					
			Method 2A compared to method 1	Method 2B 0.2M compared to method 2A	Method 2A compared to method 1	Method 2B 0.4 M compared to method 2A	Method 2A compared to method 1	Method 2B 0.4 M compared to method 2A
**Metabolites Confirmed Using Standards**								
Malate	133.05	9.6	UP(9.4)	N	N	N	N	N
Glutamate	146.04	6.2	N	N	N	N	N	DN (3.1)
PEP	166.97	14.6	N	UP (1.5)	N	N	N	N
3PGA	184.98	14.1	UP(4.1)	UP (2.6)	UP(8.4)	UP (3.5)	N	UP (5.3)
Citrate	191.02	14.8	UP(1.73)	N	UP(2.5)	UP (1.8)	N	UP (6.5)
L-Cystine	239.06	17.3	N	UP (1.6)	N	N	N	N
G6P	259.02	9.6	N	N	UP(2.2)	DN (1.6)	N	N
S7P	289.03	10.0	UP(1.72)	N	N	N	N	N
RuBP	308.98	14.9	N	UP (87.9)	N	UP(58.4)	N	UP (62.0)
UMP	323.03	11.3	UP(3.4)	N	N	N	N	DN (1.7)
Sucrose	341.11	1.7	N	N	N	N	N	DN(1.9)
IMP	347.06	11.5	N	N	N	N	N	N
ADP	426.02	14.6	UP(1.84)	UP (3.3)	N	UP(1.7)	N	UP (4.5)
ATP	505.99	15.0	UP (2.7)	N	N	UP (5.1)	N	UP (7.7)
UDP-glucose	565.05	12.2	UP (1.9)	N	N	DN (1.6)	DN (4.5)	N
ADP-glucose	588.08	13.4	UP (2.6)	N	N	DN (1.5)	N	N
Acetyl-CoA	808.09	15.5	UP (2.1)	DN(3.6)	N	N	N	N
**Multimer Adducts**								
PEP Dimer	334.91	14.7	N	UP (1.6)	N	N	N	N
3PGA dimer	370.92	14.2	UP (4.5)	UP (3.9)	UP (17.4)	UP (6.6)	N	N
3PGA trimer	556.97	14.2	UP (5.3)	UP (4.7)	N	UP (16.4)	N	N
RuBP dimer	618.96	14.9	N	UP (77.4)	N	N	N	N
ADP dimer	853.06	14.6	N	UP (11.7)	N	N	N	N
**Putative Fatty Acids**								
Dodecanoic	199.17	17.8	N	UP (2.0)	N	UP(2.5)	N	DN (1.8)
Myristic	227.21	18.3	DN(2.0)	UP (13.2)	DN (2.6)	UP(23.3)	N	UP(3.7)
Linoleic	279.23	18.7	DN(2.1)	UP (1.9)	DN (2.1)	UP(2.0)	N	N
Pentadecanoic	241.21	18.6	DN(4.4)	UP (1.9)	N	UP(3.6)	N	N

^a^UP (Fold Change) = Increased intensity with the respective fold change; DN (Fold Change): Decreased intensity with the respective fold change; N = No significant Change observed or peak not detected.

^b^Retention time shown in the table is specifically for the strain PCC 11801.

^c^Metabolites list: Malate (Mal); Glutamate (Glu); Phopshoenolpyruvate (PEP); 3-Phosphoglyceric acid (3 PGA); Citrate (Cit); L-Cystine; Glucose-6-phosphate (G6P); Sedoheptulose 7-phosphate (S7P); Ribulose Bisphosphate (RuBP); Uridine Monophosphate (UMP); Inosine monophosphate (IMP); Adenosine diphosphate (ADP); Adenosine triphosphate (ATP), Uridine diphosphate-Glucose (UDP-Glucose); Adenosine diphosphate-Glucose (ADP-Glucose); Acetyl Coenzyme A (Acetyl-CoA); Dodecanoic Acid, Myristic Acid, Linoleic Acid, Pentadecanoic Acid.

For the targeted set of metabolites, which was our focus towards ^13^C Metabolic flux analysis of these strains, we concluded that method 2B with 0.4 M NH_4_OH solution is essential for the strain PCC 7942. For PCC 7002. Importantly, the bisphosphates can not be detected without the use of NH_4_OH. Also, the fatty acids and their derivatives show lower extraction efficiency in (80–20 methanol-water)-chloroform system. In case of the strain PCC 11801, use of 100% methanol will not suffice and either method 2A or method 2B with 0.2 M NH_4_OH will yield reasonable extraction for a large number of metabolites for this strain. For all the three strains, bisphosphates such as RuBP show far superior extraction efficiency with NH_4_OH (Table 3) and thus the use of NH_4_OH is highly recommended for extraction of metabolites from cyanobacteria especially for ^13^C-MFA studies.

## Conclusion

In our study, the base method (method 1) which furnishes acceptable yields of metabolites critical in MFA studies in the strain PCC 7002, did not work well in two other strains of the same genus namely PCC 11801 and PCC 7942. The necessity to obtain efficient extraction in these strains led us to introduce variation in solvents used for quenching and extraction, based on the already available reports on other organisms. We observe drastic differences in the extraction efficiency for the three strains with minor changes in the solvent system. Our results show that with the addition of NH_4_OH there is a significant increase in the intensity of metabolite classes such as sugar phosphates, bisphosphates and NTPs. For the extraction of RuBP, an important metabolite in the flux analysis of photosynthetic organisms, the use of NH_4_OH is essential. Extraction of fatty acids into the aqueous layer improved with the addition of weak base although no significant improvement was observed for the extraction of organic acids and amino acids. These results were consistently observed for the targeted/confirmed metabolites and many putatively identified metabolites. However, there are many more m/z features which showed differential effect in the three strains with these extraction methods. Identification of these features and their extraction efficiencies will further distinguish the salient features of the method with respect to the strains. The methods used were not thoroughly examined for metabolites susceptible to alkali, other than Acetyl CoA which could be detected and quantitated in presence of lower concentrations of NH_4_OH. The subtle changes between method 2 and method 3 in these strains towards various classes of metabolites, which might be of interest in untargeted metabolomics, will have to be explored further. In conclusion, method 1 (100% methanol-chloroform-water) is not an optimal method for metabolite extractions from the strains PCC 7942 and PCC 11801. Our results demonstrates method 2B as the optimal method for metabolic flux analysis related studies where the quantitation of isotopologues of intracellular metabolites from central carbon metabolism is necessary. We envisage the use of this method in many other studies which focus on identification and/or quantification of these classes of metabolites.

## Supporting information

S1 Fig Comparison of the peak quality for a few metabolites extracted from strain PCC 7002 using the three extraction methods. (PDF)

S2 Fig Comparison of the peak quality for a few metabolites extracted from strain PCC 7942 using the three extraction methods. (PDF)

S3 Fig Comparison of the peak quality for a few metabolites extracted from strain PCC 11801 using the three extraction methods. (PDF)

S4 Fig Comparison of peak quality and intensity of a few representative metabolites extracted from strain PCC 11801 with the addition of 0.2 M NH_4_OH for phase separation. (PDF)

S1 Table Comparison of extraction methods on the putatively identified compounds. (PDF)

S1 File Processed data for the results shown in Table 3. (XLSX)

S2 File Processed data for the three strains under varying NH_4_OH concentrations. (XLSX)

## Abbreviations

3-PGA: 3 Phosphoglyceric acid
Acetyl-CoA: Acetyl coenzyme A
ADP: Adenosine diphosphat
ADP-glucose: Adenosine diphosphate glucose
ATP: Adenosine triphosphate
FBP: Fructose 1, 6-bisphosphate
G6P: Glucose 6-phosphate
IMP: Inosine monophosphate
PEP: Phosphoenolpyruvate
RuBP: Ribulose 1, 5-bisphosphate
S7P: Sedoheptulose 7-phosphate
SBP: Sedoheptulose bisphosphate
UDP-glucose: Uridine diphosphate glucose
UMP: Uridine monophosphate
