# Supplementary material for: An improved method for extraction of polar and charged metabolites from cyanobacteria
Source: PLoS One. 2018 Oct 4;13(10):e0204273. doi: 10.1371/journal.pone.0204273 (PMC6171824; doi:10.1371/journal.pone.0204273)
Supplement: S3 Fig — (PDF) [file pone.0204273.s003.pdf]

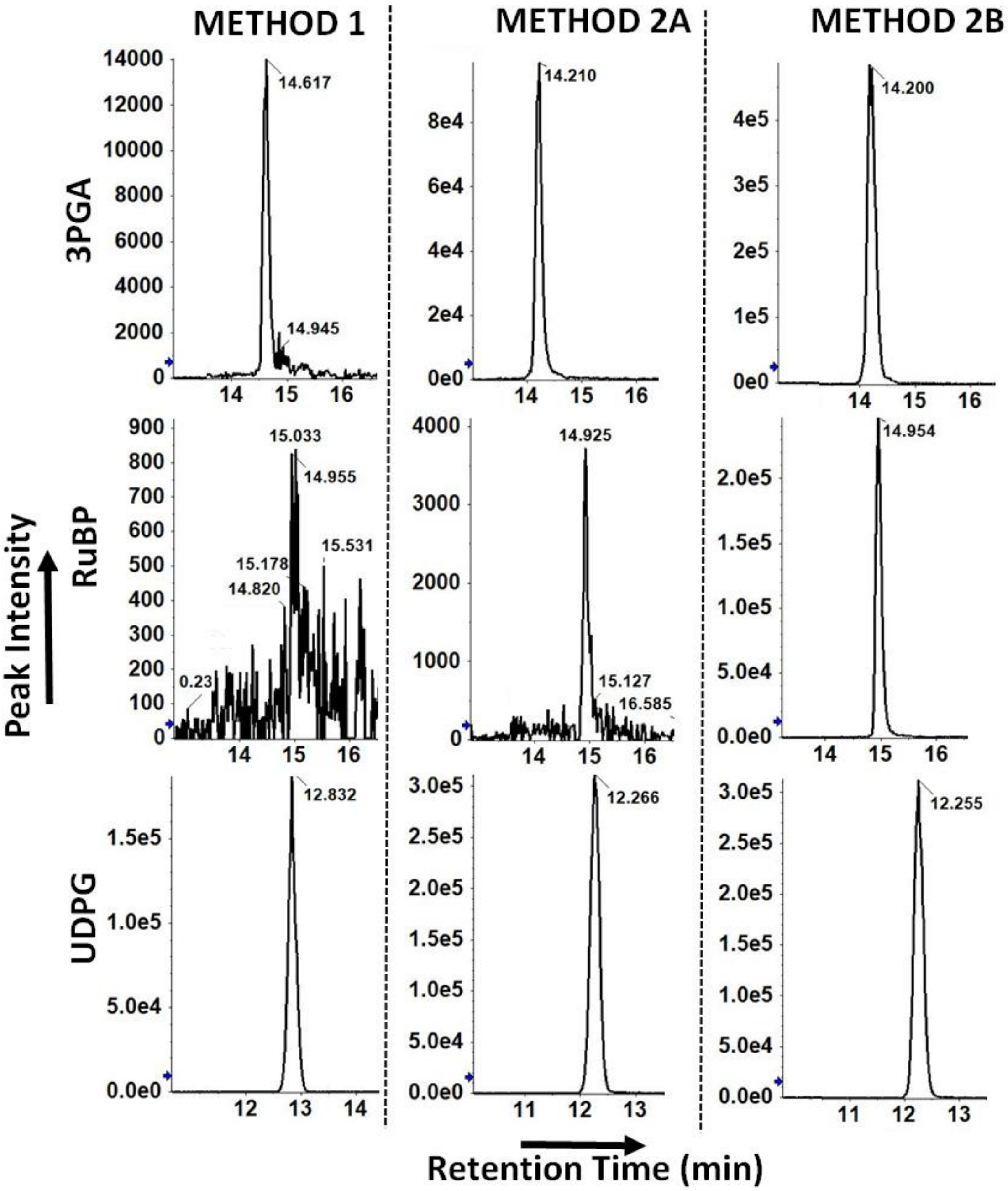

S3 Fig: Comparison of the peak quality for a few metabolites extracted from strain PCC 11801 using the three extraction methods. The extracted ion chromatogram (XIC) of the metabolites 3-PGA, RuBP, and UDPG, which are significant in the ^{13}C-MFA studies of photosynthetic organisms, obtained using the three methods are shown here to compare the extraction efficiency. The effect of addition of NH_4OH is prominent with metabolites such as RuBP and is significant for metabolites such as 3-PGA. Few other metabolites such as UDPG did not have significant change with the addition of NH_4OH. The metabolites presented are 3 Phosphoglyceric acid (3 PGA), Uridine diphosphate glucose (UDPG), and Ribulose 1,5-bisphosphate (RuBP).
