# Supplementary material for: An improved method for extraction of polar and charged metabolites from cyanobacteria
Source: PLoS One. 2018 Oct 4;13(10):e0204273. doi: 10.1371/journal.pone.0204273 (PMC6171824; doi:10.1371/journal.pone.0204273)
Supplement: S4 Fig — (PDF) [file pone.0204273.s004.pdf]

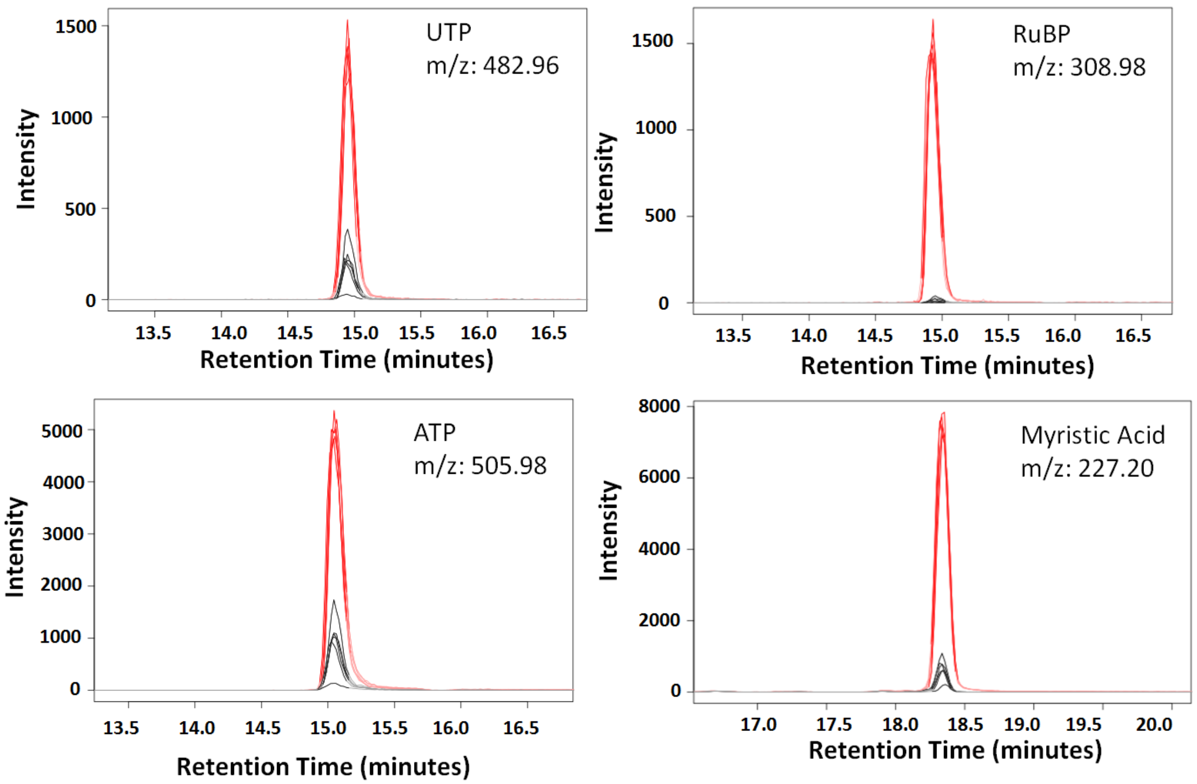

S4 Fig: Comparison of peak quality and intensity of a few representative metabolites extracted from strain PCC 11801 with the addition of 0.2 M NH_4OH for phase separation. The black trace represents the XIC of the respective metabolite obtained using method 2A and red trace represents the XIC obtained using method 2B. The peak quality of metabolites representing the classes of compounds that had significant improvement in their extraction using method 2B is shown here. The plots are obtained from pairwise comparisons of data using XCMS Online. The metabolites presented are Myristic Acid, Adenosine triphosphate (ATP), Uridine triphosphate (UTP), and Ribulose 1,5-bisphosphate (RuBP).
