# Supplementary material for: An improved method for extraction of polar and charged metabolites from cyanobacteria
Source: PLoS One. 2018 Oct 4;13(10):e0204273. doi: 10.1371/journal.pone.0204273 (PMC6171824; doi:10.1371/journal.pone.0204273)
Supplement: S1 Table — (PDF) [file pone.0204273.s005.pdf]

**S1 Table: Comparison of extraction methods on the putatively identified compounds.** Effect of quenching with methanol-water mixture (method 2) and addition of $NH_4OH$ during phase separation (method 3) on metabolites which are putatively identified based on METLIN database.

| Metabolite List | m/z | RT[b] | *Synechococcus* sp. PCC 11801 | | *Synechococcus elongatus* PCC 7942 | | *Synechococcus* sp. PCC 7002 | |
|---|---|---|---|---|---|---|---|---|
| | | | [a]Observed changes in the intensity with respective fold change | | | | | |
| | | | Method 2 vs. Method 1 | Method 3 vs. Method 2 | Method 2 vs. Method 1 | Method 3 vs. Method 2 | Method 2 vs. Method 1 | Method 3 vs. Method 2 |
| **Putative Identification using METLIN database** | | | | | | | | |
| Lactate | 89.02 | 7.5 | D(1.2) | D(1.1) | D(2.2) | N | N | N |
| Proglutamic Acid | 128.03 | 4.4 | D(1.3) | U(1.0) | N | N | N | N |
| 2-Aminooctanoic Acid | 158.12 | 16.8 | U(10.2) | N | N | U(4.6) | N | N |
| Acotinic Acid | 173.01 | 14.7 | N | U(1.0) | N | U(1.4) | D(1.1) | U(1.5) |
| 2-isopropylmalate | 175.03 | 11.4 | D(1.2) | D(3.3) | N | N | N | N |
| N-Acetyl L-Glutamic acid | 188.05 | 11.7 | N | D(1.2) | N | N | N | N |
| Dihydrobiopterin | 238.1 | 16.5 | U(2.1) | D(1.0) | N | U(2.4) | N | N |
| 2-Deoxyglucose-1 Phosphate | 245.04 | 6.7 | U(3.04) | D(1.0) | N | N | N | N |
| Glu-Leu | 259.12 | 11.9 | U(1.4) | D(1.0) | U(1.0) | D(1.6) | N | N |
| Trp-Glu | 332.12 | 14.0 | N | D(1.6) | U(1.7) | D(1.7) | N | N |
| Lithocholic Acid | 375.29 | 19.7 | D(3.4) | U(1.3) | N | U(1.2) | D(2.3) | U(3.3) |
| Cholic Acid | 407.27 | 19.4 | D(1.0) | D(1.0) | D(2.2) | U(1.9) | D(1.8) | U(2.0) |
| UDP-Xylose | 535.04 | 11.7 | U(2.4) | U(1.2) | N | N | N | N |
| GDP-gulose/GDP-galactose/GDP-Mannose | 604.07 | 11.8 | N | U(1.0) | N | N | N | N |
| UDP-N-Acetyl-D-galactosamine | 606.07 | 12.2 | D(1.2) | D(1.0) | U(1.1) | D(1.5) | N | N |
| ADP-Ribose 2 Phosphate | 638.02 | 15.1 | N | U(2.8) | N | N | N | N |
| UDP-N-acetylmuramoyl-L-alanine | 749.13 | 14.8 | N | D(1.2) | N | D(1.5) | D(1.1) | U(1.2) |

[a]UP (Fold Change) = Increased intensity with the respective fold change; DN (Fold Change): Decreased intensity with the respective fold change; N=Peak not detected.

[b]Retention time shown in the table is specifically for the strain PCC 11801.
